# Coronal Hypospadias: An Assessment of Surgical Treatment Outcomes

**DOI:** 10.34763/jmotherandchild.20252901.d-25-00032

**Published:** 2025-11-27

**Authors:** Andrzej Kowal, Monika Szelemetko, Diyaa Alhashlamoun, Ewa Sawicka

**Affiliations:** Department of Pediatric and Adolescent Surgery, Institute of Mother and Child, Warsaw, Poland

**Keywords:** coronal hypospadias, urethra, penis, prepuce, reconstruction, complications

## Abstract

**Introduction:**

A urethral meatus located in the region of the coronal sulcus accounts for approximately 25% of all hypospadias cases. Numerous surgical methods exist for the correction of this penile anomaly. According to the literature, the complication rate for this treatment ranges from 2% to 25%.

**Objective:**

The aim of this study is to assess the outcomes of coronal hypospadias treatment in boys operated on using a surgical method developed at the Department of Paediatric and Adolescent Surgery, Institute of Mother and Child.

**Material and methods:**

Between 2005 and 2023, 265 boys, aged 14 to 20 months, underwent surgery for coronal hypospadias. The operative technique involves a longitudinal incision of the dorsal part of the urethral meatus, parallel incisions of the glans, and the placement of a mattress suture encompassing the glans and the urethral apex bilaterally in the midline. Subsequently, any ventral penile curvature is corrected, and the frenulum, the normal shape of the prepuce (foreskin), and the penile skin are reconstructed. In all operated boys, a follow-up assessment was conducted at least one-year post-surgery, evaluating the location of the external urethral meatus, penile curvature, the appearance of the prepuce and penile skin, and the presence of any urethrocutaneous fistulas.

**Results:**

Of the 265 operated boys, a correctly positioned external urethral meatus was observed in 254. No patient was found to have penile curvature. The shape of the prepuce and skin was deemed normal in 256 patients. Re-reconstruction of the urethra was required in 8 patients due to meatal retraction and in 3 patients due to a fistula. The postoperative observation period was a minimum of one year.

**Conclusions:**

Based on the results of this study and a comparison with literature data, it can be concluded that the surgical method for treating coronal hypospadias developed at the Department of Surgery, Institute of Mother and Child, yields good functional results and a satisfactory cosmetic appearance of the penis.

## Introduction

Hypospadias is the most common congenital anomaly of the penis, occurring, according to various authors, in 1 per 1,000 to 4 per 1,000 live births [1]. The urethral meatus is located on the ventral surface of the penis at varying distances from its normal position, ranging from the glans to the perineum. The abnormal meatal opening is accompanied by a hooded prepuce (dorsal hood) and, in approximately 45% of cases, penile curvature (chordee). In coronal (subcoronal) hypospadias, the urethral meatus is situated near the coronal sulcus. The incidence of this meatal location is estimated at 25%, making it one of the most frequently operated types of this anomaly.

Since the 1990s, the Department of Surgery at the Institute of Mother and Child has employed a standardized method for treating coronal hypospadias [2], which is a modification of the Duckett procedure (MAGPI) [3]. The advantage of this technique, besides positioning the urethral meatus in its correct anatomical location, is the relatively straightforward possibility of performing glanuloplasty, penile skin plasty, and reconstruction of the frenulum and preputial continuity.

## Objective

The aim of this study is to assess the outcomes of coronal hypospadias treatment in boys operated on using the surgical method developed at the Department of Paediatric and Adolescent Surgery, Institute of Mother and Child.

## Material and methods

**Figure 1.**
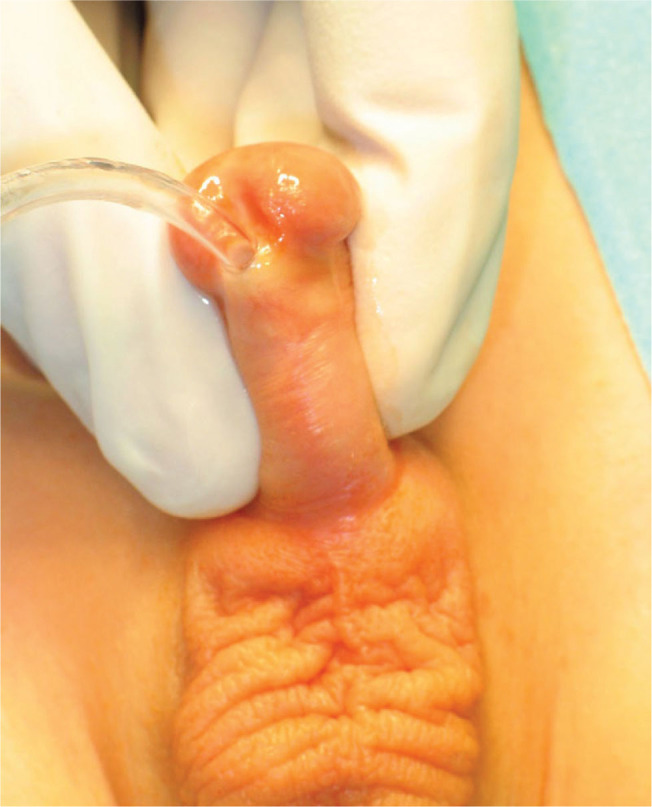
Evaluation of meatus in coronal localisation.

A urethral meatus in the vicinity of the coronal sulcus was the primary qualification for this surgical method [Fig. 1]. Between 2005 and 2023, 265 boys aged 14 to 20 months were qualified for coronal hypospadias surgery. The mean age at the time of surgery was 16 months. All patients presented with a hooded prepuce. Ventral penile curvature was diagnosed in 26 patients (9.81%).

**Figure 2.**
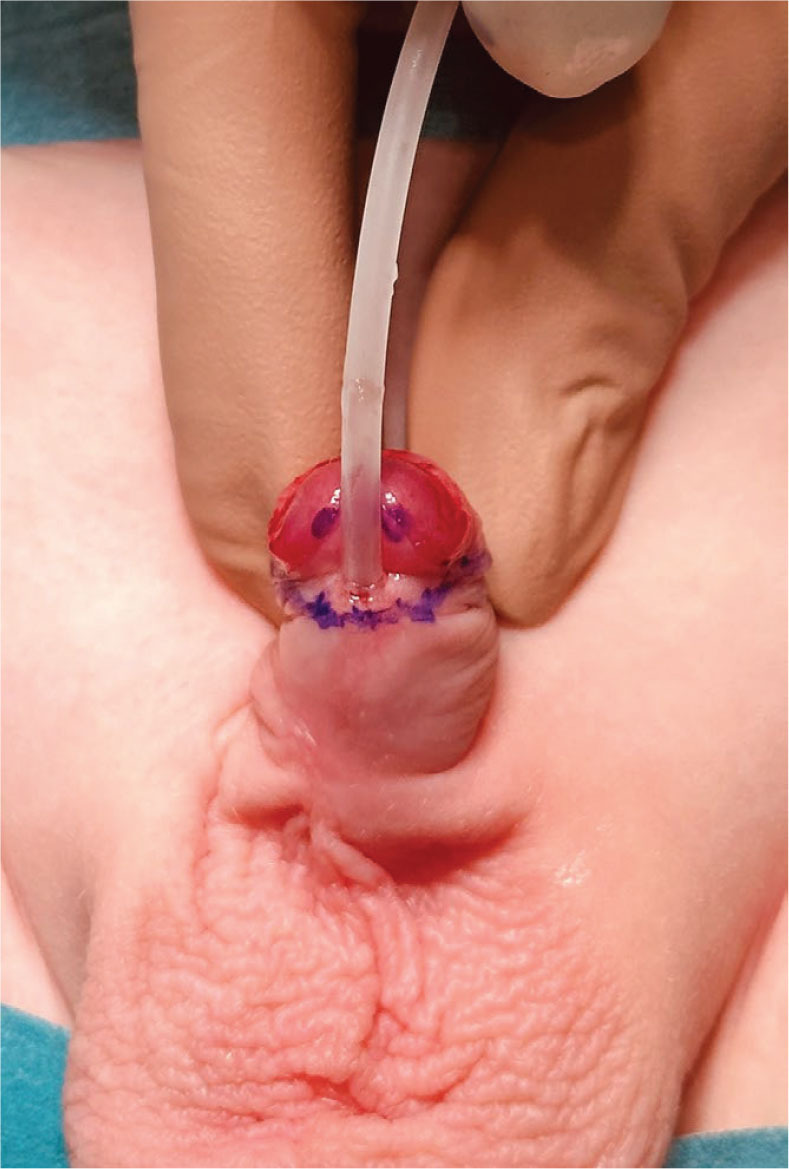
Marking points and the cutting lines to the tops of split glans and foreskin.

**Figure 3.**
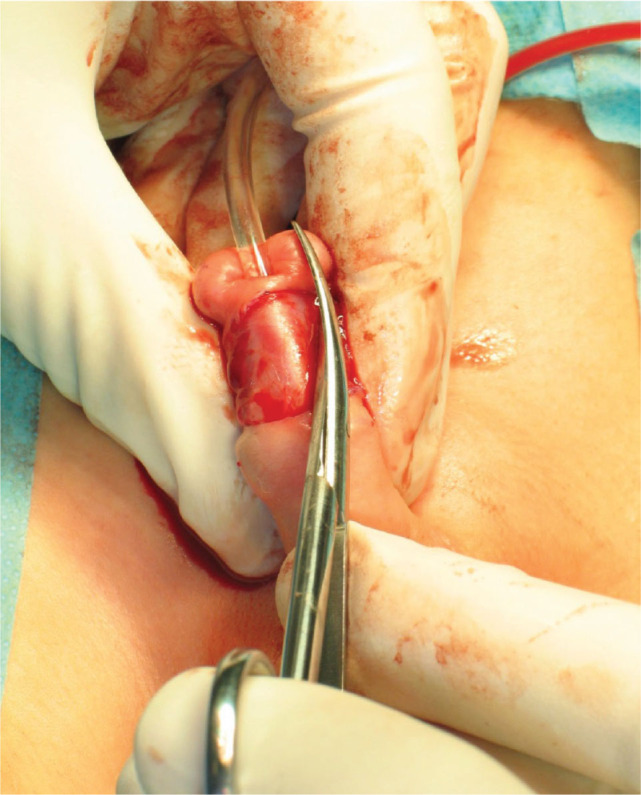
Meatoplasty procedure. Dissection of penis skin. Release abnormal fascial attachments. Vertical glans incisions.

**Figure 4.**
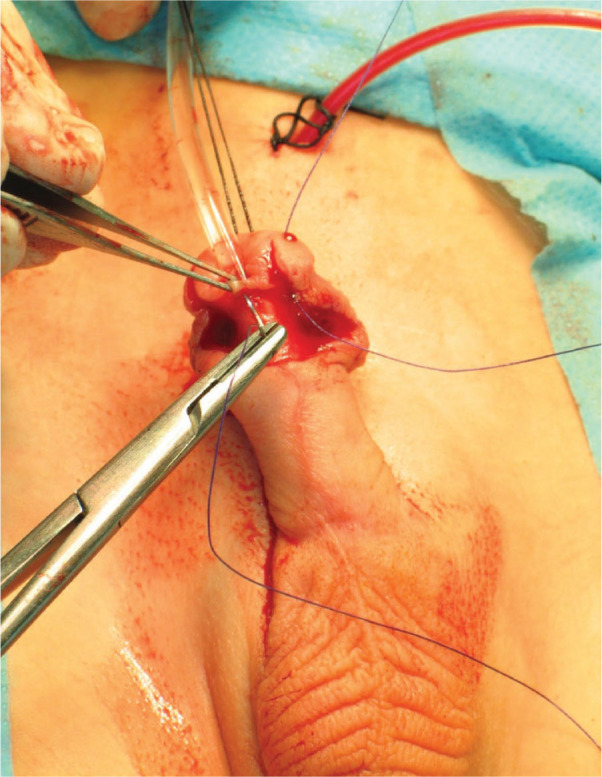
Blanket suture. Top of incision-centre of the urethra-across top of incision.

**Figure 5.**
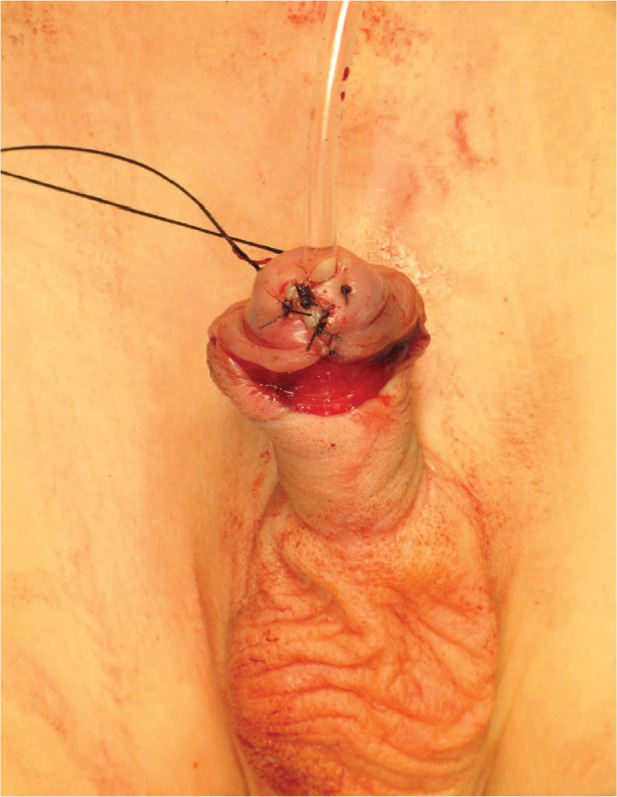
Glans blanket sutures.

**Figure 6.**
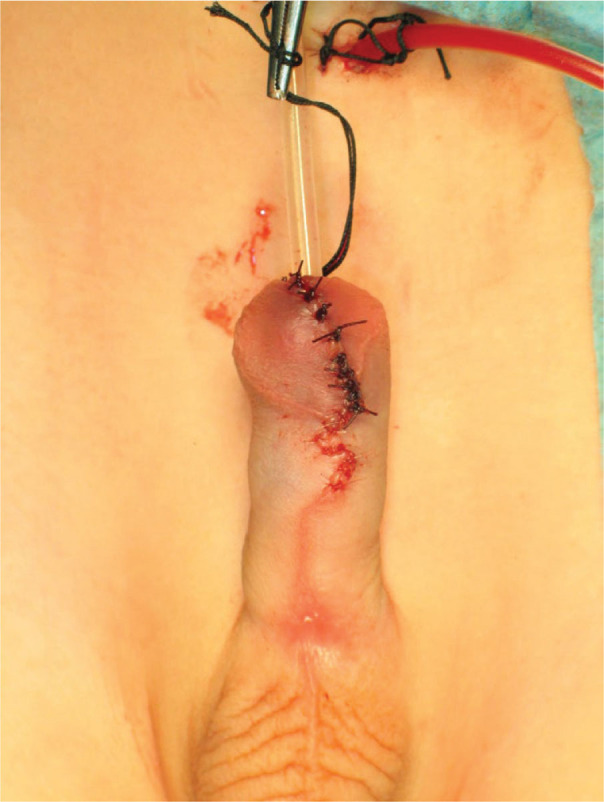
Plastic surgery of the penis frenulum, inner and outer plate the foreskin. Plastic surgery of the penis skin.

Prior to the procedure, a dorsal penile nerve block was administered. The prepuce was then retracted and cleaned. Subsequently, a size 10 French Nelaton catheter was inserted into the bladder (until 2012, suprapubic urinary diversion with a Cystofix was used). Incision lines were marked towards the apexes of the split glans and the hooded prepuce [Fig. 2]. To facilitate skin dissection, a solution of physiological saline was injected into the area of the planned incisions. A midline, vertical incision was made on the dorsal aspect of the urethral meatus, extending to the deepest point of the navicular fossa, and the wound was closed horizontally with interrupted sutures. Next, a transverse incision was carefully made to separate the skin from the ventral surface of the urethra, avoiding urethral injury. The layers of the prepuce were then incised and separated. Subsequently, the abnormal, cleft attachments of the superficial penile fascia were released. Scissors were introduced on both sides of the dissected urethra, and the glans was incised to the previously marked points [Fig. 3]. The ventral side of the urethra was connected to the glanular edge with the first mattress suture [Fig. 4]. Three mattress sutures were used to close the glans, ensuring haemostasis [Fig. 5]. The superficial penile fascia was closed with interrupted sutures. Finally, the frenulum of the prepuce was reconstructed, and the hooded prepuce was repaired, with its mobility confirmed. The penile skin was sutured [Fig. 6], and a dressing was applied.

Postoperatively, patients received furazidin at a dose of 5–7 mg/kg body weight for two days. The catheter was removed on the third postoperative day. Patients were discharged home after a physiological micturition was observed. Early treatment outcomes were assessed up to 30 days post-surgery, dividing this period into hospitalisation (3 days) and outpatient care. Late assessment was performed one year after the surgery. In the early period, the local condition (proper wound healing, appearance of the penis, and potential complications such as catheter blockage, oedema, or haematoma at the surgical site) and general symptoms (difficulty urinating, abnormal urinary stream) were evaluated. The late assessment focused on the appearance of the penis (curvature, cosmetic state of the skin scar, preputial repair), the position of the urethral meatus (potential meatal retraction), and the presence of any urethrocutaneous fistulas.

## Results

Among the 265 boys with coronal hypospadias, early complications were noted in 26 patients (9.8%). These included catheter-related problems in 8 patients (3.01%): blockage of the suprapubic drainage tube in 5 patients (1.88%) and premature removal of the Nelaton catheter in 3 patients (1.13%). Haematoma and oedema of the surgical site requiring a dressing change occurred in 18 patients (6.79%). Of these 18 boys, 6 experienced difficulty urinating (2.26%) [Table 1]. Within 30 days of surgery, the haematoma and oedema of the prepuce and penis resolved. No further difficulties in urination were observed.

**Table 1.** Early complications after coronal hypospadias treatment.

	Study participants

	265
**Early complications during hospitalisation:**	
Catheter problems, including:	8 (3.01%)
*- suprapubic urinary drainage tube obstruction*	5 (1.88%)
*- premature catheter removal*	3 (1.13%)
Haematoma and swelling requiring dressing change	18 (6.79%)
**Early complications after hospitalisation:**	
Persistent haematoma and swelling	9 (3.39%)
Dysuria	6 (2.26%)

The final assessment was conducted one year post-surgery. Of the 265 operated boys, a correct positioning of the external urethral meatus was found in 254 (95.85%). No patient exhibited penile curvature. The shape of the prepuce, its closure, and the penile scar formation were considered normal in 256 patients (96.6%) [Table 2]. Urethral re-reconstruction due to meatal retraction was required in 8 boys (3.01%), due to a urethral fistula in 3 boys (1.13%), and due to preputial dehiscence in 4 boys (1.51%) [Table 3].

**Table 2.** Late complications after coronal hypospadias treatment.

	**Study participants**

	265
**Late complications:**	
Abnormal morphology of the foreskin (separation, atrophy, asymmetry)	9 (3.39%)
Retraction of the external urethral meatus	8 (3.01%)
Urethral fistula	3 (1.13%)

**Table 3.** Late complications after coronal hypospadias surgery requiring surgical treatment.

	**Study participants**

	265
**Late complications:**	
Abnormal morphology of the foreskin (separation, atrophy, asymmetry)	4 (1.51%)
Retraction of the external urethral meatus	8 (3.01%)
Urethral fistula	3 (1.13%)

## Discussion

Many surgical methods for hypospadias repair have been described and are in use. The goals of surgery are to reconstruct the missing urethral segment and position it at the tip of a well-formed glans, straighten the penis, and achieve a satisfactory cosmetic appearance of the prepuce and the skin covering the penis. This paper aims to summarize the results of coronal hypospadias treatment using the surgical method developed at the Department of Surgery, Institute of Mother and Child. Since 1987, coronal hypospadias operations were performed at our department using the method proposed by Duckett [3]. After these operations, the external urethral meatus did not reach the tip of the glans. Complications prompted the search for new solutions. Similar approaches were proposed by Arap (1984, M conf.) [4], Zaontz (1989, GAP) [5], Decter (1991, M inv. V glans.) [6], Harrison and Grobbelaar (1997, UGPI) [7], and a very similar method by Gilpin, Clements, and Boston (1993, GRAP) [8]—the only one that included preputial reconstruction.

Our method is distinguished by the first mattress suture that connects the ventral surface of the urethral meatus to the edges of the incised glans, anchoring and creating the meatus at the glans tip. Equally important is the meticulous reconstruction of the penile fascia, correction of curvature, and reconstruction of the frenulum and preputial skin. The fundamental principle of our developed method is to restore the normal anatomical state of the penis. Rebuilding all the appropriate layers covering the urethra prevents fistula formation. Other authors also emphasize the importance of a two-layer closure over the neourethra [9,10,11]. Restoring the anatomical continuity of the fascial layers on the ventral side facilitates preputial reconstruction and maintains its mobility. Careful separation of the outer and inner layers of the prepuce allows for an appropriate preputioplasty, ensuring a normal appearance of the entire operated organ. This is highlighted by the authors of many studies [8,12,13,14]. In Poland, great importance is attached to the presence of the prepuce [15]. Our experience shows that parents prefer the preservation of a normal prepuce whenever possible [16]. The discussed method allows for the reconstruction of this important anatomical detail.

Despite the many (approximately 250) described and applied original and modified surgical methods, delicate surgical techniques, new surgical sutures, and advances in postoperative care, complications after coronal hypospadias surgery are still reported, with an incidence estimated from 2% to 25% [17].

Van der Werff [18] categorizes complications based on the need for additional surgery into three grades: Grade I – minor complications not requiring surgery (e.g., minor wound dehiscence, haematoma, urinary retention); Grade II – cosmetic complications requiring relative reoperation (e.g., abnormal prepuce appearance, excessive penile scarring); Grade III – serious or functional complications requiring absolute reoperation (e.g., fistula, stenosis, penile curvature).

Bhat and Mandal [19] analysed early complications based on articles from PubMed. For distal hypospadias, the acceptable rate of all early complications was below 5%, but haematoma and penile oedema were observed in about 10% of operated boys by Alaraby [20] and in 11% by Nonomura [21], while in our material, it was 6.79% [Table 1]. Preputial dehiscence or loss was noted in 9 of our operated boys (3.39%), whereas in a meta-analysis by Yuhao et al. [22], wound dehiscence was described in 2.1% of patients. Interestingly, their results showed no difference in the incidence of urethral fistulas between patients circumcised during surgery and those who underwent preputial reconstruction.

The most severe complication requiring reoperation is a urethral fistula. In coronal hypospadias, this complication is estimated to occur in 1% to 16.7% of cases [17]. Urethral fistula formation is secondary to infection, ischemia, suture line tension, or distal stenosis. The low number of fistulas in our patients—1.13% [Tables 2, 3]—was comparable to other authors [17]. A meta-analysis of complications in non-proximal hypospadias was performed by Yuhao et al. [22]. They summarized 44 studies and 10,666 cases. In this vast material, the fistula rate was 4%.

In their analysis, the meatal retraction rate was estimated at 3.4%. In our material, the higher number of meatal retractions (3.01%) compared to urethral fistulas (1.13%) [Tables 2, 3] may be related to the imprecise placement of the first mattress suture connecting the urethra to the glans. Problems with meatal retraction were reported by Decter et al. [6], where the rate of this complication was 12.5% in 16 operated boys. All our patients with a urethral fistula and meatal retraction required reoperation [Table 3]. Additionally, 4 patients were reoperated for necessary preputioplasty.

Two types of ventral penile curvature accompanying hypospadias are distinguished. The first type, where chordee is occasional, occurs in patients with distal hypospadias (so-called “skin chordee”) [23]. A superficial, subcutaneous chordee proximal to the urethral meatus is corrected by mobilizing the skin below and releasing the abnormal attachments of the superficial fascia. The second type affects patients with more severe forms of hypospadias. Snodgrass reported a 15% rate of penile curvature in distal hypospadias surgeries [24]. In our material, this was 9.81%. Patient and thorough release of the abnormal fascial attachments allowed for the correction of ventral curvature in all patients.

The results of surgical treatment for coronal hypospadias using the method developed at the Department of Surgery, Institute of Mother and Child, are good. The method allows for the restoration and reconstruction of all abnormalities present in this type of hypospadias. It enables the creation of a urethral meatus in the correct location and achieves an appropriate appearance of the prepuce and penis. The low number of complications advocates for the use of this method in selected and strictly qualified cases of hypospadias, specifically coronal hypospadias.
